# Effect of silymarin on the relative gene expressions of some inflammatory cytokines in the liver of CCl_4_-intoxicated male rats

**DOI:** 10.1038/s41598-023-42250-7

**Published:** 2023-09-14

**Authors:** Sarah M. El-Kot, Wessam Wanas, Afaf M. Hafez, Nihal A. Mahmoud, Amina M. Tolba, Abeer H. Younis, Gamal El Sayed, Huda E. Abdelwahab

**Affiliations:** 1 Biochemistry Department, Faculty of Science, Alexandria University, Alexandria, 21568 Egypt; 2 Environmental Studies Department, Institute of Graduate Studies and Research, Alexandria University, Alexandria, 21526 Egypt; 3 Materials Science Department, Institute of Graduate Studies and Research, Alexandria University, Alexandria, 21526 Egypt; 4 Physiology Department, Faculty of Medicine (Girls), Al-Azhar University, Cairo, 11754 Egypt; 5 Anatomy Department, Faculty of Medicine (Girls), Al-Azhar University, Cairo, 11754 Egypt; 6 National Institute of Oceanography and Fisheries (NIOF), Alexandria, 21556 Egypt; 7 Waste Water Lab, Baheria Water and Waste Company, Baheria, Damanhur, 107 Egypt; 8 Chemistry Department, Faculty of Science, Alexandria University, Alexandria, 21568 Egypt

**Keywords:** Biochemistry, Biomarkers

## Abstract

The intensive exposure of the liver cells to any type of noxae, such as viruses, drugs, alcohols, and xenobiotics could induce hepatic inflammation through the upregulation of gene expression of several fibrotic and inflammatory mediators. So, our study assessed the role of silymarin on the inflammatory response induced by carbon tetrachloride (CCl_4_) as an example of xenobiotics on liver tissues in male rats. Forty-eight Wister male rats (weight: 130 ± 10) were housed for 14 days and then divided randomly into six groups: control, SLY: rats received only silymarin orally for 12 weeks (daily), CO: rats were injected with corn oil for 8 weeks (3 times weekly), CCl_4_: rats were injected with CCl_4_ solubilized in corn oil for 8 weeks (day by day), Treated: rats received silymarin for 4 weeks after CCl_4_ injection, Protected: rats received silymarin for 4 weeks before and 8 weeks during CCl_4_ injection. When the treatment period for the rats was over, they underwent scarification after anesthesia. Then, the sera were extracted from the collected blood for the determination of irisin levels, liver functions, and lipid profiles. Liver tissues were separated for the histopathological examinations, the determination of oxidative stress (OS) parameters content, and the relative gene expression of inflammatory cytokines; nuclear factor kappa (NF)-κB, tumor necrosis factor-alpha (TNF-α), interleukin (IL)-6, cyclooxygenase (COX)-2, and transforming growth factor beta (TGF-β). The findings showed that silymarin reduced liver inflammation by overcoming the OS process and inflammatory cytokines production which was stimulated by CCl_4_. These results were confirmed by histopathology of liver tissues.

## Introduction

The liver is the biggest digestive gland in the body, not only responsible for the metabolism of carbohydrates, proteins, and lipids, but also has a role in the secretion and biotransformation. It has a great capacity to detoxify toxic substances and produces useful end products^1,2^. Acute Liver injury can be caused by excess alcohol consumption or exposure to hepatotoxins and can progress to severe hepatic diseases such as hepatitis, cirrhosis, and cancer^3^. Acute Liver injury is linked to oxidative stress (OS) that produces harmful intermediates such as free radicals and redox-active reactants^4^. It can then progress to acute liver failure, which develops into hepatic encephalopathy and multiple organ failure, with a high mortality rate^5^.

The most widely used animal model for hepatic fibrosis and cirrhosis is the rat with repeated exposure to carbon tetrachloride (CCl_4_)^6^. CCl_4_ is one of the most potent environmental contaminants manufactured chemically. Humans are exposed to CCl_4_ via oral, inhalation, and dermal routes. It is a precursor for chlorofluorocarbon gases that have been used as aerosol propellants^7^. It mediates hepatotoxicity after its biotransformation by the hepatic microsomal cytochrome (CYP) 450 into trichloromethyl (CCl_3_^·^) radicals. These free radicals initiate cell steatosis, thereby causing the impairment of liver function^8,9^. CCl_3_^·^ free radicals attack the lipids in the membrane of the cell and some organelles, including mitochondria and endoplasmic reticulum, initiate the peroxidation process and alter the permeability of these compartment membranes that cause cell necrosis^10^. Those radicals could also attack membrane proteins and thiols. In addition, CCl_4_ caused the activation of Kupffer cells to secrete large amounts of inflammatory factors that exacerbate inflammation^11^. Hepatocyte necrosis is associated with the release of cellular components, including ATP, mitochondrial DNA, and certain molecules, which activates the phosphorylation of nuclear factor kappa (NF)-κB. This results in the production of pro-inflammatory mediators (e.g., cyclooxygenase (COX)-2 and interleukin (IL)-6)). That activation ends after the secretion of pro-fibrogenic cytokines that can induce hepatic fibrogenesis^11^.

Irisin is a metabolic hormone, that biosynthesizes from the cleavage of the extracellular part of the transmembrane fibronectin type 3 domain-containing (FNDC)-5^12^. It is supposed to be involved in the maintenance of metabolic homeostasis. It has been first identified as a myokine that is controlled by the peroxisome proliferator-activated receptor gamma coactivator 1 alpha (PGC1α) and exercise^13^. In adipose tissues, irisin encourages the browning of white adipose tissue to enhance energy expenditure, which could limit gain in body weight and insulin resistance^14^. In myocytes, it increases fatty acid oxidation and glucose utilization^15^. In hepatocytes, it inhibits gluconeogenesis, lipogenesis, and lipid accumulation^16^. It was also found that irisin possesses strong antioxidant properties so a negative relationship between irisin levels and OS was expected. While hepatocytes express irisin, the association between systemic irisin levels and hepatic injury is still unknown^17^.

Herbal drugs are used on a large-scale around the world because of their action against many diseases and intoxication^18^. Silymarin is a safe mixed herbal metabolite that is used as an antifibrotic, antioxidant, and anti-inflammatory against hepatotoxicity^19^. Silymarin extract is derived from Silybum marianum and consists of flavonolignans (silydianin, silychristin, silybin, and iso silybin) with small quantities of flavonoids (taxifolin), polyphenolics, and fatty acids that have a wide spectrum of metabolic regulatory properties^20^. Silybin is the main bioactive component of silymarin and was the first member to be discovered in 1959 as a new family of flavonolignans^21^. It is believed to possess antioxidant properities and prevent lipid oxidation in human and some animal models^22^.

The protective mechanisms of silymarin have not been precisely evaluated. Thus, the principal aim of the existing research is to introduce a novel study to assess the possible protective mechanisms of silymarin against hepatic damage in rats subjected to CCl_4_, by mapping the relationship between the irisin hormone, oxidative stress and inflammation.

## Materials and methods

### Chemicals and materials

Silymarin (Legalon 140™) was obtained from Rottapharm-Madaus Co. (Cairo, Egypt). Corn oil was gotten from the united oil processing and packing company S.A.E (El-Sharkya, Egypt). CCl_4_ (purity grade, 99.9%) was obtained from Sigma-Aldrich (St Louis, MO, USA). Kits of Thiobarbituric acid reactive substances (TBARS), nitric oxide (NO), glutathione (GSH), glutathione-S-reductase (GSR), glutathione-S-transferase (GST), glutathione peroxidase (GPx), superoxide dismutase (SOD), Serum total protein (TP), alkaline phosphatase (ALP), alanine aminotransferase (ALT), aspartate aminotransferase (AST), triglycerides (TG), and high-density lipoprotein cholesterol (HDL-c) were obtained from Biodiagnostic (Cairo, Egypt). Irisin kit was purchased from Cono Biotech Co. Ltd. (China). Biozol component was bought from Invitrogen (CA, USA). Primers for NF-κB, COX-2, IL-6, TNF-α, and the transforming growth factor beta (TGF-β) were obtained from Bioneer (Korea). SYBER Green 1-step Kit was obtained from Thermo Scientific (USA).

### Methods

All experiments were performed in accordance with relevant guidelines and regulations.

#### Animals

**Figure 1:**
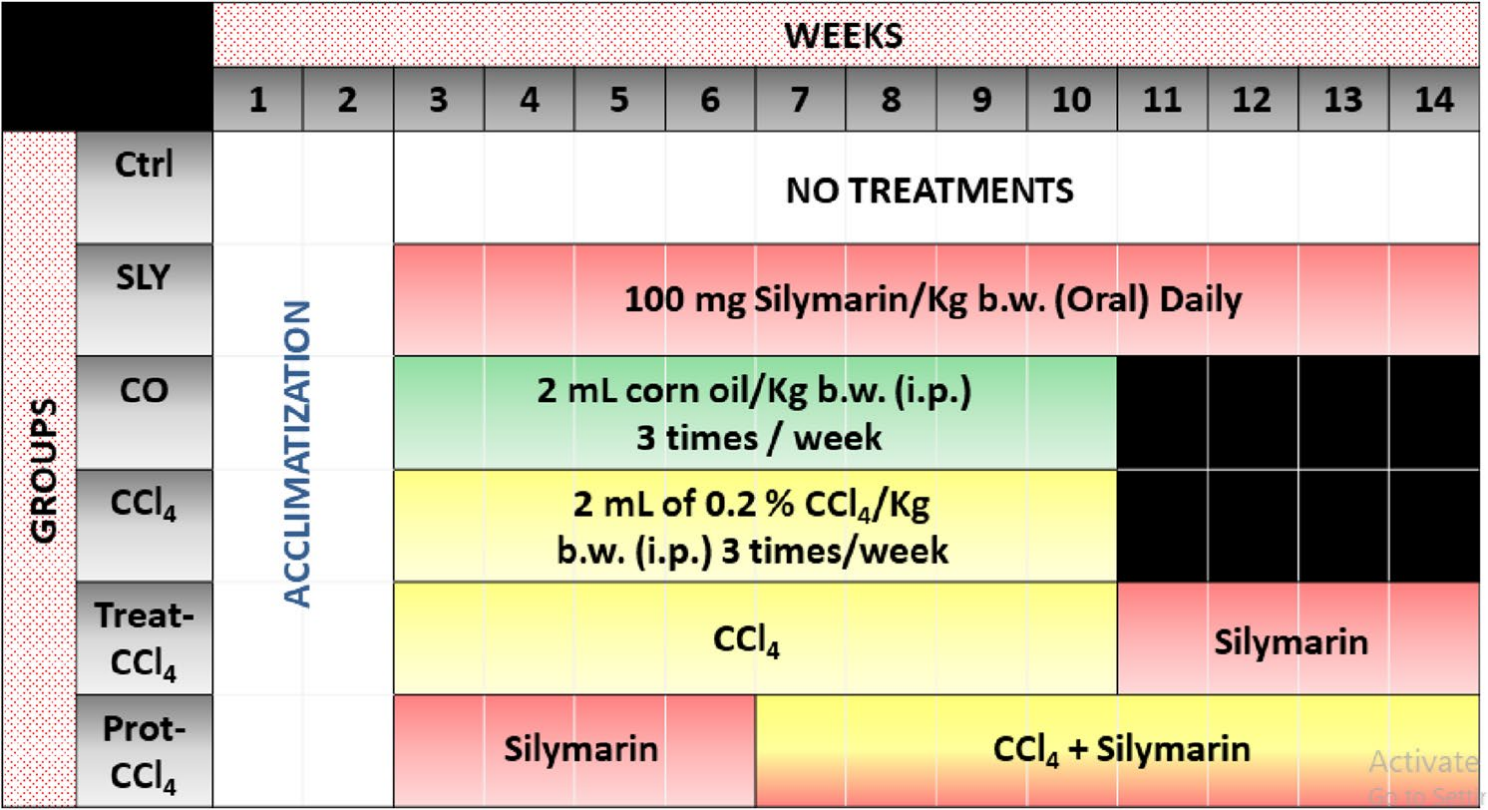
Experimental design flowchart.

Forty-eight male Wister rats, whose ages ranged from 60 to 65 days (130 ± 5 g), were purchased from the animal room of the Faculty of Agriculture, Alexandria University. They were kept in suitable cages with environmentally relevant circumstances (temp. 25 ± 0.5 °C, 12:12 sleep/wake phase). Rats were fed on a common pellet diet and tap water ad libitum^23,24^. They were left for 2-week adaptation before starting the treatments. Figure 1 resembles the experimental design.

In brief, rats were distributed randomly into 6 groups, (8 rats/each) as bellow:

1. *Control group (Ctrl)* healthy rats with no treatment.
2. *SLY* rats were administered with silymarin (100 mg/Kg body weight) orally every day for 3 months^25^.
3. *Vehicle group (CO)* rats were injected with corn oil (2 mL/Kg) intraperitoneally (i.p.) 3 times weekly for 2 months.
4. *Induced group (CCl*_*4*_*)* rats were injected with 2 mL of 0.2% CCl_4_ (dissolved in corn oil)/Kg (i.p.) 3 times weekly for 2 months^23,26^.
5. *Treated group (Treat-CCl*_*4*_*)* rats were administered silymarin 4 weeks after stopping the CCl_4_ injection (as in the CCl_4_ group).
6. *Protected group (Prot-CCl*_*4*_*)* rats were given silymarin 4 weeks pre and 8 weeks during the CCl_4_ injection.

When the experiment period was completed, feeding was stopped for 12 hs, and the rats were anesthetized by inhalation with 5% isoflurane. After dissection, blood samples were gathered from the portal vein and underwent centrifugation at 1000×*g* over 10 min; the separated sera were reserved in a deep freezer until analysis. Additionally, livers were detached rapidly and divided into three pieces after washing with cold saline. The first piece of the liver was bored by a needle and fixed (in 10% formalin/saline) for histological examinations. The next piece was transmitted to vials containing RNA later solution and saved at − 80 °C until the process for the detecting the relative gene expression of fibrotic and inflammatory mediators. The last portion was homogenized with the homogenizer in cold sodium phosphate buffer/saline solution and centrifuged at 10,000×*g* over 20 min. The supernatants were distributed over some vials to avoid sample freezing and thawing and stored at − 80 °C till use for the oxidative and nitrosative stress (OS/NSS) markers detection^23,24^.

#### Ethics statement

The ethical standards of the animal protocol have been certified by the Institutional Animal Care and Use Committee of Alexandria University (ALEXU-IACUC; Code No. AU14-220,529-3-2). This work was carried out following ARRIVE guidelines (https://arriveguidelines.org).

### Histological, biochemical, and molecular examinations

#### Histopathological examinations of liver tissues

Liver pieces were transferred to 10% formalin/Saline solution, fixed, embedded in paraffin, and then sliced into 5 μm thickness Sections^27^. The grade of histological changes in liver tissue was performed by hematoxylin and eosin (H&E) dye and Gomori’s Trichrome (fibrosis detector) staining. All slides underwent examination and imaging using a light microscope.

#### Determination of the hepatic oxidative and nitrosative stress (OS/NSS) parameters

Markers of OS/NSS were assessed in the liver tissue homogenate supernatant using commercial kits. Lipid peroxidation, the peroxidation and decomposition of polyenic fatty acids of the lipids, was assessed following the method of Ellman^28^. TBARS, including lipid aldehydes, and hydroperoxides, are predicted in terms of malondialdehyde (MDA) equivalents. Under high temperatures and low PH, MDA reacts with thiobarbituric acid, giving a pinky solution, which is detected at 532 nm. The concentration of NO, an indicator of NSS, was valued after adding of Griess reagent to the homogenate^29^. The level of GSH was performed according to the method of Ellman^30^. Ellman’s reagent reacts with GSH, producing a yellow product, which is observed at 412 nm. The SOD activity was assessed by the indirect method using pyrogallol, as cited by Marklund and Marklund^31^. At high PH, pyrogallol autooxidation takes place spontaneously, forming superoxide anion radicals (O_2_^−^·), which are removed by SOD. The GPx activity was evaluated spectrophotometrically using hydrogen hydroperoxide as a substrate^32^. The GR catalyzes the production of GSH from the oxidized form GSSG in the presence of NADPH^33^. Glutathione-S-transferase (GST) activity was valued based on the modified methodology of Habig et al*.*, since the p-nitrobenzyl chloride (GST substrate) reacts with the GSH forming a conjugate product detected at 310 nm^34^. The hepatic total protein in the homogenate was estimated to calculate of enzyme activity based on the Lowry et al*.* method^35^.

#### Biochemical Analysis of irisin, Liver Enzymes, and Lipid Profile in serum

Irisin was measured in the rat’s serum by commercial ELISA kits^12^. Liver enzymes (TP, ALT, AST, and ALP) and lipid profiles (HDL, LDL, and TG) were measured using commercial kits.

#### Assessment of the hepatic inflammatory and fibrotic mediators’ relative gene expression

The relative gene expressions of NF-κB, COX-2, TNF-α, IL-6, and TGF-β were assessed by the reverse transcription-polymerase chain reaction (qRT-PCR). The isolation of the total RNA from the frozen liver tissues (nearly 50 mg) was performed using Biozol reagent according to Shaban et al^23^. The mixture was treated with chloroform and centrifuged at 4 °C over 20 min. The aqueous layer was collected, and the ethanol was added to precipitate the mRNA. The purity was assessed at the absorption ratio of 260/280 nm, which was regularly higher than 1.8. Using a one-step RT-PCR reaction, the cDNA was synthesized from 5 µg of the purified mRNA by the High-Capacity cDNA Reverse Transcription Kit and used for amplification of transcripts primer sequences. qRT-PCR was done using 3 µL of isolated RNA in a 30 µL reaction mixture, which was contain 0.25 µM of the requested gene primer (listed in Table 1) and 12.5 µL Sybr Green real-time PCR. Every run consisted of 50 °C over 2 min and 95 °C over 10 min, subordinated by 45 cycles of 95 °C over 15 s, 60 °C over 20 s, and 72 °C over 60 s in the PCR instrument (Stratagene, Agilent Biosciences). The expression of all the genes were standardized to that of β-actin mRNA in the same liver sample, and the fold difference was calculated using the Eq. 2^−ΔΔ^Ct, as described before^23,36,37^.

**Table 1:** qRT-PCR primers sequences.

Name	Sequence	Name	Sequence
β-actin	F:5′-AGCCATGTACGTAGCCATCC-3′	IL-6	F:5′-AGTTGCCTTCTTGGGACTGA-3′
	R:5′-CTCTCAGCTGTGGTGGTGAA-3′		R:5′-ACAGTGCATCATCGCTGTTC-3′
NF-kB	F:5′-ACGATCTGTTTCCCCTCATCT-3′	TGF-β	F:5′-CTTTGCTCATGGCAGTACATCTG-3′
	R:5′-TGCTTCTCTCCCCAGGAATA-3′		R:5′-CCTTTAACAACATCCCGATTCC-3′
TNF-α	F:5′-AGATGTGGAACTGGCAGAGG-3′	COX-2	F:5′-CTGTATCCCGCCCTGCTGGTG-3′
	R:5′-CCCATTTGGGAACTTCTCCT-3′		R:5′-TTGCGTTGATGGTGGCTGTCTT-3′

### Statistical analysis

Data were provided as mean ± standard deviation (SD). Results were analyzed for statistical variances using one-way analysis of variance (ANOVA). A *p* value ≤ 0.05 was marked statistically significant. Comparisons between groups were performed using the post hoc analysis by SPSS 25.0 software. Pearson correlation coefficient was applied to parametrical data. Still, Spearman rank correlation coefficient was performed on the non-parametrical data to determine the association between irisin hormones and all parameters in all groups.

## Results

### Silymarin overcomes the changes induced by CCl_4_ in liver architecture

**Figure 2:**
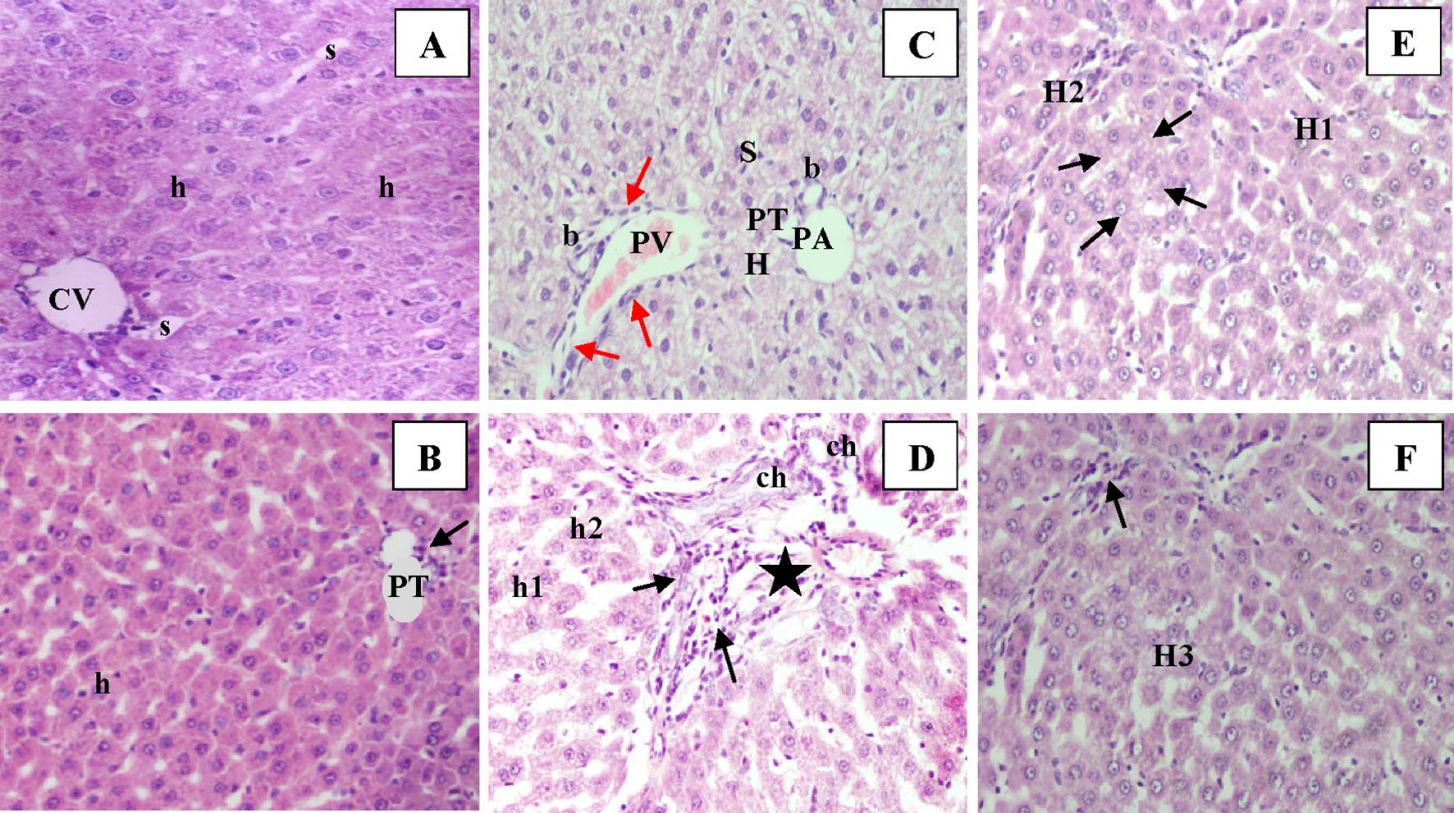
Microscopic examination of liver tissues stained by H&E; (**A**) Ctrl group: control rat liver demonstrating the normal architecture of the central compartment of the hepatic lobule. The central vein (CV) occupies the core of the lobule. Strings of hepatocytes arrayed from the CV. They are departed by narrow sinusoids (s). The hepatocytes demonstrate a normal granular and eosinophilic cytoplasm. Hepatocytes have a single nucleus (h); (**B**) SLY group: Liver section from a rat receiving silymarin for 12 weeks revealing the unaltered organization of cords of hepatocytes radiating around the CV and focal aggregates of cellular infiltrates (arrow) are depicted around the portal tract (PT); (**C**) CO group: The PT of the Oil-treated rat liver demonstrating vacuolation of the hepatocyte cytoplasm (H). the portal vein (PV) tributary, the branch of the portal artery (PA), (b) = bile ductules, scanty stromal cells (arrow); (**D**) CCl_4_ group: The PT of the liver of rat injected with CCl_4_ induced lesion revealing consistent increased connective tissue stroma (black star) with dilation of biliary cholangioles (ch). Some hepatocytes have a single nucleus (h1); others are binucleated (h2). Pivotal clumps of cellular infiltrate (arrow) are depicted. (**E**) Treat-CCl_4_ group: Treatment with silymarin after CCl_4_-induced lesion depicting focal areas (arrows) with vacuolated hepatocytes (H1) among the majority of normal hepatocytes (H2). (**F**) Prot-CCl_4_ group: Liver section of rat treated with silymarin before and during CCl_4_ injection; showing recovery of hepatocytes (H3) and general architecture of the hepatic lobule. All images are original with microscopic magnification × 400.

**Figure 3:**
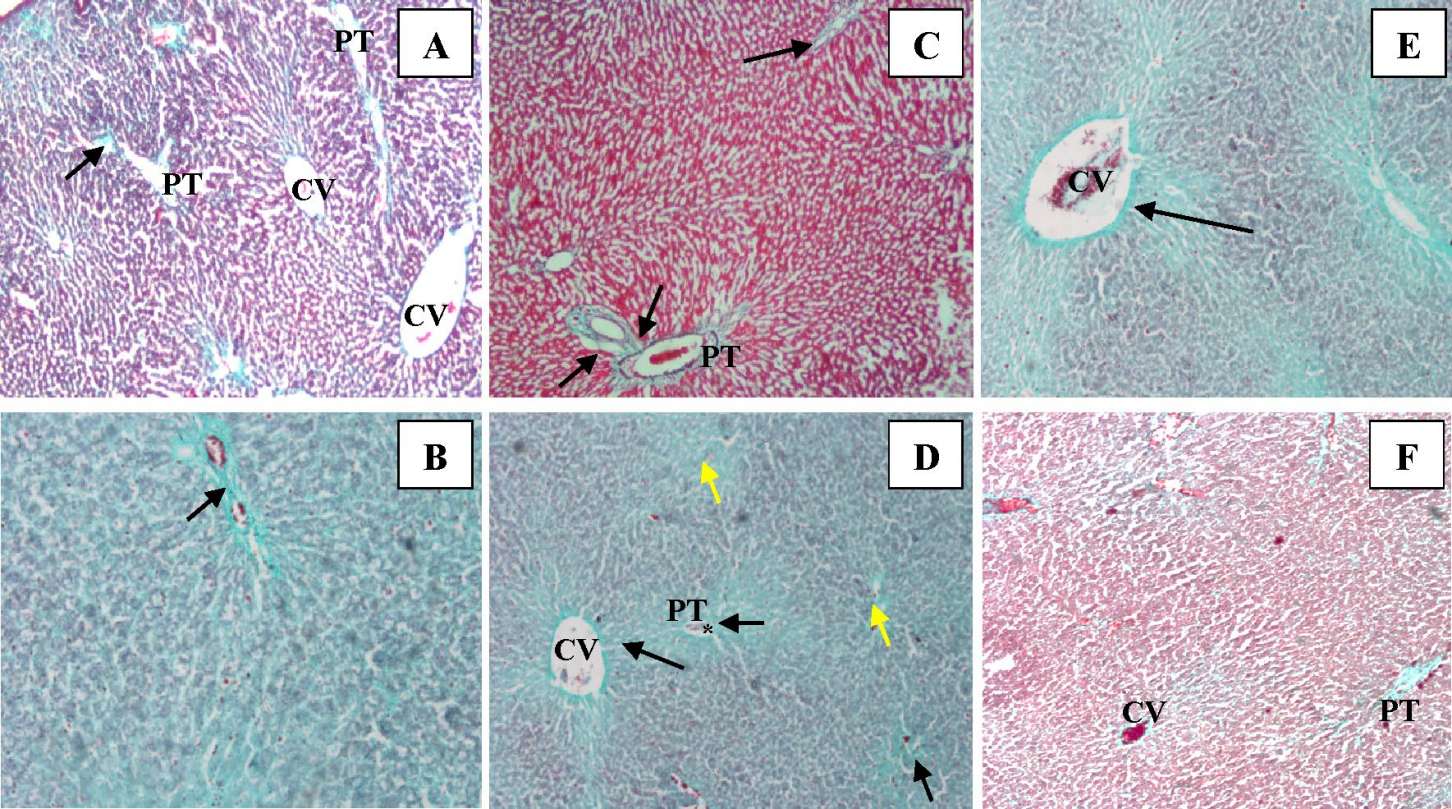
Microscopic examination of liver tissue stained by Gomori’s trichrome stain; (**A**) Ctrl group: Negative control rat liver demonstrates the supporting stroma and minimal collagen fiber content (arrow) depicted in the PT. (**B**) SLY group: Liver section from a rat receiving silymarin for 12 weeks showing average connective tissue content (arrow pointing to greenish collagen fibers) (**C**) CO group: liver of rat treated with oil vehicle showing average connective tissue deposit (arrow) in the PT (**D**) CCl_4_ group: Liver section from rat treated with CCl_4_ induction of lesion illustrating non-resolving of the fibrous tissue (arrow pointing to green collagen fibers) deposited in the PT and the CV. Note the extensive diffusion of degenerated cells (yellow arrow) appeared between the hepatocytes. Abnormal inspissation of erthrocytes (*) noticed in the hepatic sinusoids as in the PT tributaries. (**E**) Treat-CCl_4_ group: liver of rat treated with silymarin 4 weeks after CCl_4_ induced lesion demonstrating persisting collagen bands arrow pointing to a greenish band of fibers) around the dilated central vein. (**F**) Prot-CCl_4_ group: Liver section from rats treated with silymarin before and during CCl_4_ induced lesion demonstrating an evident reduction in the greenish deposits of collagen fibers around the CV and the PT stroma. All images are original with microscopic magnification × 100.

Examination of H&E and Gomori’s Trichrome stained sections of all studied groups existed in Figs. 2 and 3. The control and SLY groups showed that they displayed nearly identical histological pictures. Livers of the control and SLY groups showed no morphometric or histological alterations between them. The livers seemed to be composed of the normal architecture of the central compartment of the hepatic lobule. Each one was occupied centrally by a central vein. Cords of mono-nucleated hepatocytes with normal granular and eosinophilic cytoplasm radiated from the central vein (CV) departed by narrow sinusoids. Oil-treated liver (CO group) showed some binucleated hepatocytes with cytoplasm vacuolation and dilatation of blood sinusoids among hepatocytes. Additionally, the Gomori’s Trichrome stain demonstrated the supporting stroma in the liver tissue with minimal collagen fiber content, which appeared in the portal tracts (PT). Rat liver treated with CCl_4_ revealed focal areas with eosinophilic hepatocytes and zones of vacuolated pale hepatocytes in the liver tissue. Some PT shows compact cellular infiltration surrounded by fibers. There are clusters of degenerated hepatocytes without nuclei. The Gomori’s Trichrome stain demonstrated dense green assemblies of fibers between the liver lobules with vacuolated hepatocytes. It also illustrated abnormal inspissation of erythrocytes within the hepatic sinusoids as in the PT tributaries. While, Treatment with silymarin (Treat-CCl_4_) displayed regaining the eosinophilic granular existence in the hepatocytes cytoplasm, general restoration of hepatic cords arrangement, and intervening blood sinusoids with some blood congestion in the branch of the portal artery and nuclei of the cellular infiltrate in the stroma of the PT. In the protected group (Prot-CCl_4_) administration with silymarin pre and during CCl_4_ injection showed recovery of hepatocytes and general architecture of the hepatic lobule. It also reduced the deposition of collagen fibers, which was induced by CCl_4_ around the CV and in the PT stroma.

### Silymarin terminated lipid peroxidation induced by CCl_4_

The effects of silymarin on the lipid peroxidation parameters, NO and MDA, in rat liver are shown in Table 2. The NO and MDA content had non-remarkable (*p* > 0.05) changes in the hepatic homogenates of rats treated with silymarin alone (SLY group) compared to the control. The levels of those parameters in rats injected with corn oil alone (CO group) were increased significantly (*p* ≤ 0.05) compared with the Ctrl group. In addition, rats injected with CCl_4_ dissolved in corn oil (CCl_4_ group) demonstrated a significant (*p* ≤ 0.05) elevation in NO and MDA concentrations compared with the Ctrl and CO group, while the administration of silymarin in the treated and protected groups (Treat-CCl_4_ and Prot-CCl_4_) alleviated NO and MDA levels significantly (*p* ≤ 0.05) compared to the CCl_4_ group but did not achieve the control.

**Table 2:** Effects of Silymarin on OS/NSS parameters.

Parameters	Groups					
	Ctrl	SLY	CO	CCl_4_	Treat-CCl_4_	Prot-CCl_4_
NO (µM/mg protein)	4.94 ± 0.2^a^	4.43 ± 0.1^a^	10.5 ± 0.7^b^	24.5 ± 0.8^c^	14.4 ± 0.8^d^	10.8 ± 0.9^b^
MDA (nmol/mg protein)	1.71 ± 0.1^a^	1.68 ± 0.1^a^	3.14 ± 0.2^b^	6.00 ± 0.2^c^	3.39 ± 0.2^d^	1.95 ± 0.3^e^
GSH (mg/mg protein)	2.01 ± 0.0^a^	2.01 ± 0.0^a^	1.65 ± 0.1^b^	0.25 ± 0.0^c^	1.57 ± 0.0^d^	1.90 ± 0.0^e^
GR (µmol/min/mg protein)	0.02 ± 0.0^a^	0.02 ± 0.0^a^	0.03 ± 0.0^b^	0.06 ± 0.0^c^	0.04 ± 0.0^d^	0.02 ± 0.0^a^
GST (µmol/min/mg protein)	6.41 ± 0.2^a^	6.45 ± 0.2^a^	4.30 ± 0.0^b^	1.06 ± 0.0^c^	3.93 ± 0.0^d^	5.86 ± 0.1^e^
GPx (U/mg protein)	16.0 ± 0.4^a^	16.4 ± 0.2^ad^	12.0 ± 1.7^b^	4.0 ± 0.1^c^	12.3 ± 0.3^b^	16.8 ± 0.7^d^
SOD (U/mg protein)	8.98 ± 0.4^a^	9.34 ± 0.4^a^	5.53 ± 0.4^b^	3.73 ± 0.4^c^	6.44 ± 0.3^d^	7.49 ± 0.4^e^

All values are presented as mean ± SD (n = 8), Different characters with the same parameters are significantly different at *p* < 0.05*.*

### Silymarin endorsed the antioxidant system

The effects of silymarin on the antioxidant markers (GSH, GR, GPx, GST, and SOD) in rat liver are shown in Table 2. The GSH level, as well as GR, GPx, GST, and SOD activities had non-remarkable (*p* > 0.05) changes in the SLY group compared to the Ctrl group. The activity of GR was noticeably (*p* ≤ 0.05) higher while the concentration of GSH and the activities of GPx, GST, and SOD were significantly (*p* ≤ 0.05) lower in the CO group in comparison to the Ctrl group. Additionally, the CCl_4_ group demonstrated a significant (*p* ≤ 0.05) elevation in the activity of GR and a significant (*p* ≤ 0.05) decline in the level of GSH and the activities of GPx, GST, and SOD in comparison to the Ctrl and CO groups. Conversely, Treat-CCl_4_ and Prot-CCl_4_ groups demonstrated a significant (*p* ≤ 0.05) decline in the activity of GR and a significant (*p* ≤ 0.05) elevation in the level of GSH and the activities of GPx, GST, and SOD in comparison to the CCl_4_ groups but did not achieve the control (*p* ≤ 0.05).

### Silymarin treatment improved liver functions and lipid profiles

The hepatic function and lipid profile tests in the serum of different studied groups are shown in Table 3. The TP, ALT, and TG levels in the SLY group had non-significant (*p* > 0.05) changes, whereas the AST, ALP, and LDL-c levels were increased significantly (*p* ≤ 0.05), and the HDL-c level was elevated significantly (*p* ≤ 0.05) when compared with the Ctrl group. The concentrations of ALT, AST, ALP, LDL-c, and TG were noticeably (*p* ≤ 0.05) higher, while the concentrations of TP and HDL-c were significantly (*p* ≤ 0.05) lower in the CO group as compared with the ctrl group. Moreover, the CCl_4_ group showed significant (*p* ≤ 0.05) elevations in the ALT, AST, ALP, LDL-c, and TG levels, whereas the TP and HDL-c levels were considerably (*p* ≤ 0.05) declined as compared to the Ctrl and CO groups. However, the ALT, AST, ALP, LDL-c, and TG levels of Treat-CCl_4_ and Prot-CCl_4_ groups were reduced significantly (*p* ≤ 0.05). Still, the concentrations of TP and HDL-c were improved significantly (*p* ≤ 0.05) as compared to the CCl_4_ group but didn’t achieve the control.

**Table 3:** Effect of silymarin on the hepatic functions and lipid profile parameters.

Parameters	Groups					
	Ctrl	SLY	CO	CCl_4_	Treat-CCl_4_	Prot-CCl_4_
TP (g/dL)	5.7 ± 0.1^a^	5.7 ± 0.1^a^	3.4 ± 0.1^b^	2.8 ± 0.1^c^	3.1 ± 0.1^d^	4.5 ± 0.2^e^
ALT (U/L)	41.2 ± 0.4^a^	41.0 ± 0.5^a^	104.0 ± 0.7^b^	151.5 ± 0.5^c^	100.5 ± 1.4^d^	82.8 ± 1.1^e^
AST (U/L)	73.4 ± 0.4^a^	68.8 ± 1.7^b^	140.9 ± 3.8^c^	217.3 ± 0.4^d^	167.9 ± 0.7^e^	138.7 ± 0.8^f^
ALP (U/L)	90.5 ± 0.8^a^	88.5 ± 3.2^b^	187.8 ± 2.1^c^	231.2 ± 0.9^d^	167.9 ± 0.5^e^	155.8 ± 1.1^f^
TG (mg/dL)	73.8 ± 0.9^a^	73.2 ± 1.3^a^	250.6 ± 1.7^b^	325.5 ± 3.2^c^	226.0 ± 0.2^d^	273.0 ± 1.2^e^
LDL-C (mg/dL)	35.6 ± 0.9^a^	34.4 ± 1.2^b^	47.4 ± 0.8^c^	238.2 ± 0.5^d^	123.5 ± 0.8^e^	62.5 ± 0.5^f^
HDL-C (mg/dL)	51.6 ± 0.8^a^	52.4 ± 0.9^b^	33.4 ± 0.6^c^	18.4 ± 0.4^d^	35.2 ± 0.8^e^	40.7 ± 0.4^f^

All data are presented as mean ± SD (n = 8), Different characters with the same parameter are significantly different at *p* ˂ 0.05*.*

### Silymarin elevated irisin

**Figure 4:**
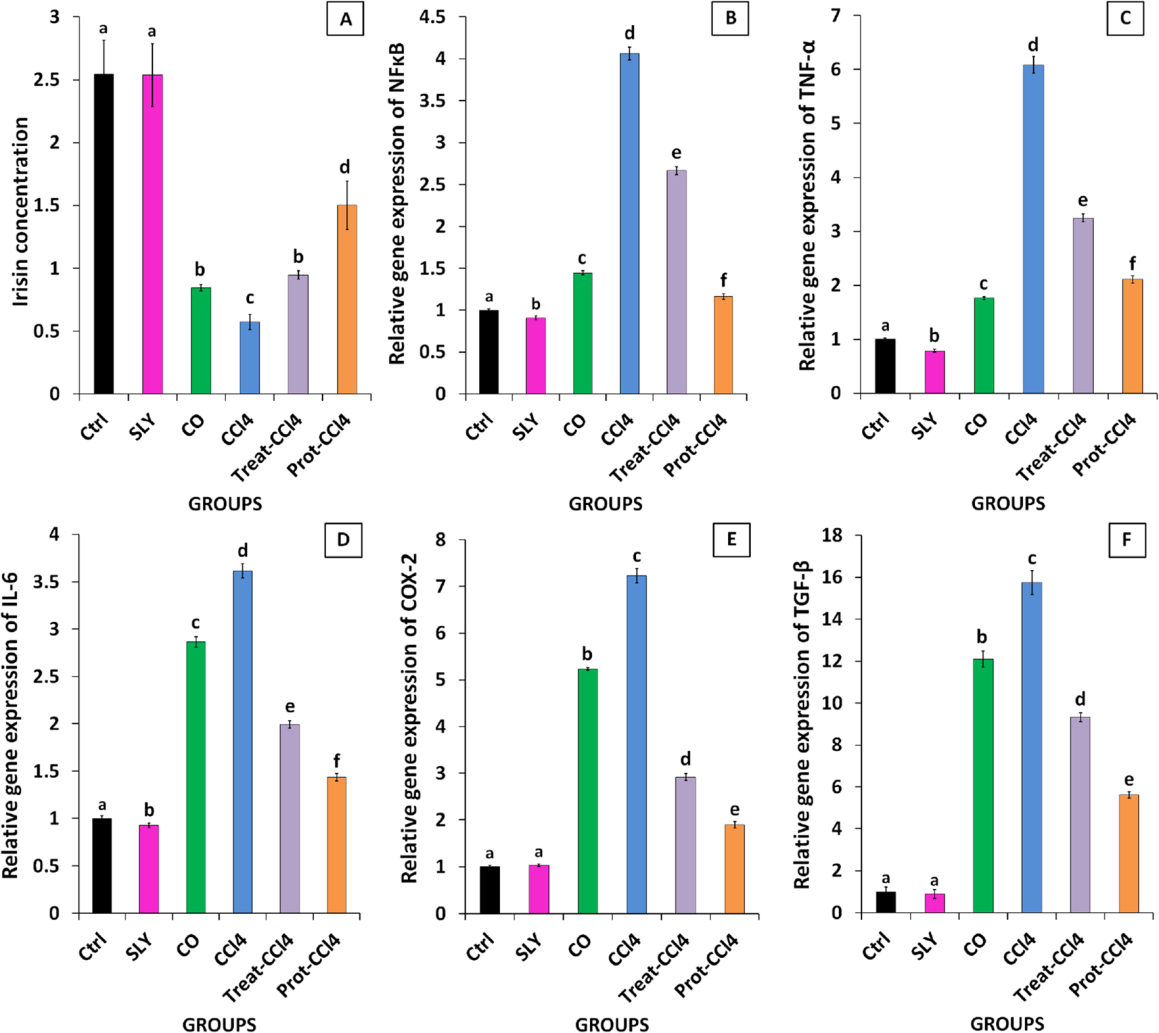
Effect of silymarin administration on (**A**) the concentrations of Serum Irisin and the gene expression of the hepatic; (**B**) NF-κB, (**C**) TNF-α, (**D**) IL-6, (**E**) COX-2, and (**F**) TGF-β against CCl_4_-injection. The findings are expressed as mean ± SD (n = 8). Different characters for the same parameter are significantly different at *p ˂* 0.05.

Irisin levels in different studied groups were presented in Fig. 4A. The concentration of Irisin in the SLY group wasn’t affected (*p* > 0.05) compared to the control. In contrast, the Irisin content was significantly (*p* ≤ 0.05) decreased in the CO group in comparison with the Ctrl. Moreover, the Irisin level was significantly (*p* ≤ 0.05) lower in the CCl_4_ group as compared to the CO and Ctrl groups. In Treat-CCl_4_ and Prot-CCl_4_ groups, silymarin administration upset the content of Irisin than the CCl_4_ group significantly (*p* ≤ 0.05) but didn’t achieve the control.

### The anti-inflammatory effects of silymarin

The impacts of silymarin on the relative gene expression of the inflammatory parameters in all studied groups are presented in Fig. 4. The NF-κB and TNF-α relative gene expressions were noticeably (*p* ≤ 0.05) lower, while the IL-6 and COX-2 relative gene expressions were noticeably (*p* ≤ 0.05) higher in the SLY group compared to the healthy group. However, the relative gene expression of those genes was significantly (*p* ≤ 0.05) higher in the CO group in comparison to the Ctrl. Also, CCl_4_ group showed significant (*p* ≤ 0.05) elevation in the NF-κB, TNF-α, IL-6, and COX-2 gene expressions as compared to CO and Ctrl groups. However, the administration of silymarin in the treated and protected groups presented a significant (*p* ≤ 0.05) decline in the gene expressions of those parameters compared to the CCl_4_ group but did not achieve the control.

### Silymarin reduced fibrosis

The relative gene expression of the fibrotic marker; TGF-β in different studied groups is shown in Fig. 4F. The TGF-β relative gene expression in the SLY group was significantly (*p* ≤ 0.05) lower than the control. In contrast, the TGF-β relative gene expression in the CO group was significantly (*p* ≤ 0.05) higher compared to the control. Moreover, the relative gene expression of that parameter in the CCl_4_ group was significantly (*p* ≤ 0.05) higher compared to the CO and Ctrl groups. In Treat-CCl_4_ and Prot-CCl_4_ groups, silymarin administration upset the fibrotic effect of CCl_4_ significantly (*p* ≤ 0.05) but didn’t achieve the control.

### Correlation analysis between Irisin and OS/NSS parameters, inflammatory, and fibrotic markers in all studied groups

The correlation analysis is presented in Table 4. Results showed that there was a strong positive association between the serum level of Irisin and the TP (*r* = 0.873*, p* < 0.01), HDL-c (*r* = 0.962, *p* < 0.01), the hepatic GSH (*r* = 0.891, *p* < 0.01), GST (*r* = 0.879, *p* < 0.01), GPx (*r* = 0.787, *p* < 0.01), SOD (*r* = 0.958, *p* < 0.01) and the relative gene expression of the hepatic TNF-α (*r* = 0.761, *p* < 0.05) in the control group. But, a strong negative association was observed between the serum level of Irisin and ALP (*r* = − 0.949, *p* < 0.01), ALT (*r* = − 0.951, *p* < 0.01), AST (*r* = − 0.841, *p* < 0.01), LDL-c (*r* = − 0.789, *p* < 0.01), and TG (*r* = − 0.785, *p* < 0.01), the hepatic MDA (*r* = − 0.887, *p* < 0.01) and GR (*r* = − 0.827, *p* < 0.01), and the relative gene expression of NF-κB (*r* = − 0.893, *p* < 0.01), IL-6 (*r* = − 0.928, *p* < 0.01), COX-2 (*r* = − 0.949, *p* < 0.01), and TGF-β (*r* = − 0.942, *p* < 0.01) in the control group. A strong negative correlation between the serum level of Irisin and TP (*r* = − 0.761, *p* < 0.05), and the relative gene expression of the hepatic COX-2 (*r* = − 0.850, *p* < 0.01) was noticed in the CO group. Additionally, A strong positive association was detected between the serum level of Irisin and ALP (*r* = 0.753, *p* < 0.05), and the hepatic level of GST (*r* = 0.807, *p* < 0.05) in the CCl_4_ group. Conversely, a strong negative association was observed between the serum level of Irisin and the hepatic MDA (*r* = − 0.862, *p* < 0.05), the relative gene expression of the hepatic NF-κB (*r* = − 0.946, *p* < 0.05), and TNF-α (*r* = − 0.922, *p* < 0.05) in the CCl_4_ group. It was identified that there was a strong positive correlation between the serum level of Irisin and HDL-c (*r* = 0.709, *p* < 0.05) in the Treat-CCl_4_ group. A strong negative association was observed between the serum level of Irisin and the LDL-c (*r* = − 0.770, *p* < 0.05), TG (*r* = − 0.957, *p* < 0.01), and the relative gene expression of the hepatic IL-6 (*r* = − 0.772, *p* < 0.05) in the Treat-CCl_4_ group. Additionally, a strong negative association was noticed between the serum level of Irisin and TG (*r* = − 0.736, *p* < 0.05) in the Prot-CCl_4_ group. Further, no more significant correlations were observed.

**Table 4:** Correlation analysis between Irisin hormone and all studied parameters in all studied groups (n = 8).

Parameters	Groups					
	Ctrl	SLY	CO	CCl_4_	Treat-CCl_4_	Prot-CCl_4_
Irisin	1	1	1	1	1	1
TP	0.873^b^	− 0.050	− 0.761^a^	− 0.552	− 0.244	− 0.075
ALP	− 0.949^b^	− 0.080	− 0.241	0.753^a^	0.209	0.247
ALT	− 0.951^b^	− 0.052	− 0.193	0.572	0.235	− 0.430
AST	− 0.841^b^	0.144	− 0.299	0.132	0.482	− 0.075
HDL-c	0.962^b^	0.520	0.663	0.217	0.709^a^	0.161
LDL-c	− 0.789^b^	− 0.521	− 0.491	− 0.241	− 0.770^a^	0.384
TG	− 0.785^b^	− 0.256	0.060	0.176	− 0.957^b^	− 0.736^a^
NO	− 0.113	− 0.515	− 0.183	0.085	− 0.156	0.506
MDA	− 0.887^b^	0.067	− 0.358	− 0.862^b^	− 0.307	− 0.561
GSH	0.891^b^	0.207	− 0.218	− 0.386	0.120	0.327
GR	− 0.827^b^	− 0.319	− 0.228	0.303	− 0.307	0.442
GST	0.879^b^	− 0.037	0.506	0.807^a^	0.319	0.175
GPx	0.787^b^	− 0.255	− 0.347	0.367	0.623	− 0.024
SOD	0.958^b^	0.035	− 0.399	0.012	0.685	0.667
NF-κB	− 0.893^b^	− 0.276	0.363	− 0.946^b^	− 0.527	− 0.659
TNF-α	0.761^a^	− 0.052	0.695	− 0.922^b^	0.551	− 0.293
IL6	− 0.928^b^	0.531	− 0.315	0.000	− 0.772^a^	0.494
COX-2	− 0.958^b^	− 0.086	− 0.850^b^	0.108	0.602	− 0.196
TGF-β	− 0.942^b^	− 0.195	− 0.659	0.635	0.602	− 0.538

Where; ^a^ Correlation is significant at the 0.05 level (2-tailed), ^b^ Correlation is significant at the 0.01 level (2-tailed).

#### Experimental summary

**Figure 5:**
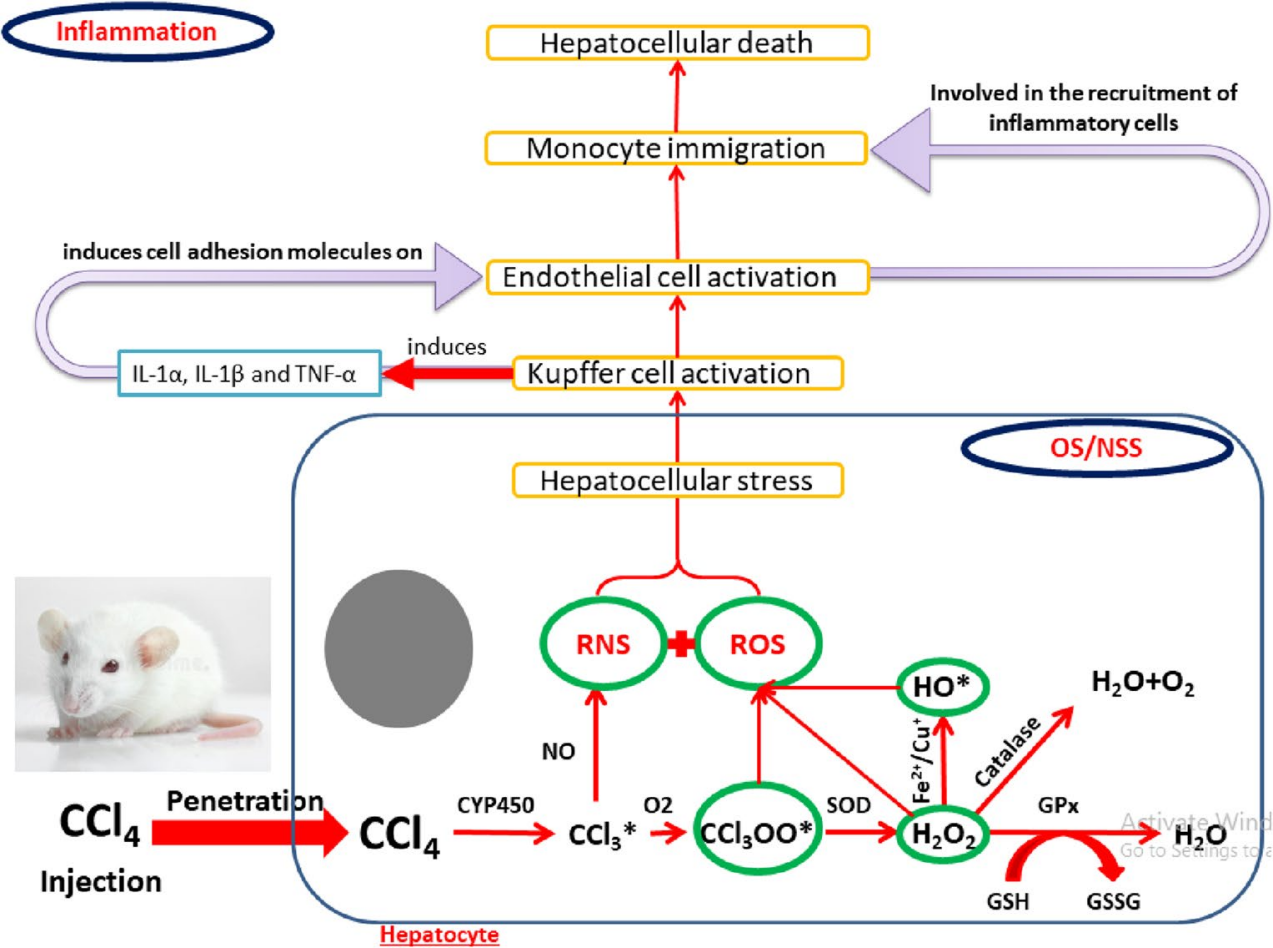
Graphical abstract.

All results of the present experiment were concise in Fig. 5.

## Discussion

The current study purveys evidence for the OS/NSS, which is progressed by CCl_4_. The levels of MDA and NO were increased significantly in liver homogenates of rats in both CO and CCl_4_ groups (Table 2). In addition, the antioxidant system was also interrupted by rigorous depletions of the GSH backup and the GPx, GST, and SOD activities with an obvious increase in GR activity (Table 2). Previous studies showed that the corn oil administration causes significant elevation in liver MDA level with a significant decline in GSH level, SOD and GPx activities^38–40^. They recommended that animals administrated with corn oil (containing a high amount of polyunsaturated fatty acids) have low scavenging activity to diminish free radicals and more predisposed to lipid peroxidation than animals fed with monounsaturated fats or saturated fatty acids diets^40^. As mentioned before, CCl_4_ promotes the production of various free radical metabolites, which induce the overproduction and accumulation of ROS (reactive oxygen species) and enhance the β-oxidation of polyunsaturated fatty acids of the liver cell phospholipid bilayer consequently increasing the level of MDA^9,23^. Reactive nitrogen species (RNS) are also elevated because the metabolites of CCl_4_ activate the inducible nitric oxide synthase (iNOS) activity, the key enzyme for the excessive production of NO. The depletion of GSH concentration is the main step in the initiation of hepatic necrosis. The GSH was consumed by the two enzymes; GPx and GST to detoxify CCl_4_ and its metabolites, which explains the reason for GSH deficiency. The increment of GR activity may be considered a natural response to compensate for the continuous depletion of GSH backup. The accumulation of ROS may cause damage to the catalytic activity of SOD. The enzyme activity of t-GPx and GST may possibly inhibit due to the depletion in the GSH level and the direct attack of the free radicals to the enzyme active sites^23,41^. Previous studies indicated that the administration of CO with CCl_4_ amplified the hepatotoxic effects of CCl_4_^40^.

In our experiment, CO and/or CCl_4_ administration triggered an obvious increment of ALT, AST, and ALP with considerable depletion in TP concentration, which indicates liver cell damage in both CO and CCl_4_ groups (Table 3). The elevated levels of those hepatic enzymes in serum are due to the cellular leakage of those enzymes into the blood circulation which reflects the image of cell membrane integrity damage of the hepatocytes. The decline of serum TP may be due to decreasing protein biosynthesis^23,41^. In this study also, the hepatic injury altered the lipid profiles, where the LDL-c and TG concentrations were increased, whereas the HDL-c concentration decreased (Table 3). The results were approved and agreed with the last literatures which indicated that CO and/or CCl_4_ induced toxic hepatotoxicity^23,40–44^.

The CO and CCl_4_ groups showed a significant upregulation of the NF-κB, COX-2, TGF-β, TNF-α, and IL-6 gene expression in rats’ livers compared to the control (Fig. 4). OS is the primary mechanism in the induction of chronic hepatic inflammation which mainly evolved from fibrosis and progressed to liver cirrhosis. It encourages cytokine biosynthesis such as NF-κB via the provoking of Kupffer cells. NF-κB is a primary transcription factor regulating the induction of a series of pro-inflammatory cytokines gene expression and involves in the progression of inflammation and apoptosis. COX-2 is an inducible isozyme of cyclooxygenases and a key player in initiating the inflammatory response^45^. Biernacki et al. reported that the proteasomal degradation of NF-κB (p65) can terminate the pro-inflammatory cascade reaction mediated by NF-κB then stop the gene expression of several pro-inflammatory genes, COX-2, and TNF-α^46^. Rusyn et al*.,* indicated that single dose of corn oil upregulated the expression of NF-κB and TNF-α^47^. CCl_4_ enhances the activation of cytoplasmic NF-κB, which then Trans-locates to the nucleus and induces TGF-β gene expression. TGF-β activates human hepatic stellate cells and transforms them into myofibroblasts by increasing the deposition of collagen in between cells^24^. The activated NF-κB also induces the transcription of TNF-α and iNOS gene, which give us another explanation for the overproduction of NO^23,48–50^. TNF-α, is a multifunctional cytokine, initiates the up-regulation of some interleukins (IL-6, IL-8, IL-12), and other inflammatory intermediates gene expression through inflammatory cascade reactions^51,52^.

In addition, the histological examinations confirmed the biochemical results (Figs. 2C,D and 3D) where CCl_4_ influenced extensive modifications in the liver tissue architecture as severe hepatocellular degenerations, inflammatory cells, and the existence of fatty changes.

The results demonstrated that the MDA and NO levels were decreased significantly in the hepatic homogenates of rats administrated with silymarin in the protected and treated groups while the GSH concentration and the GPx, GR, GST, and SOD activities were increased (Table 2). Silymarin maintains the integrity of liver cells through several mechanisms^53^. First, it increases the plasma membrane stability by reacting with the hydrophobic compounds in the inner layer of the cell membrane. This reaction avoids the access of pro-oxidants such as CCl_4_ through the cell membrane, decreasing the rate of lipids peroxidation and decreasing MDA levels, thereby preserving the SOD catalytic enzyme activities which are affected by the free radicals. Second, the depletion in the contents of the GSH is probably antagonized by silibinin (silymarin isomer). Silibinin may increase the generation of the hepatic GSH via an increment in the substrate (i.e., cysteine) bioavailability, thereby preserving the antioxidants enzymes relay on the GSH (i.e., GSH-Px, GST) and GR enzyme activity due to the stable consumption of GSH^25^.

In the current study, silymarin decreased the elevation of ALT, AST, and ALP enzyme activities, TG and cholesterol levels with a decline of TP level in the sera which are detected after CCl_4_ injection in both treat-CCl_4_ and prot-CCl_4_ groups (Table 3). In hepatocytes, the impacts of silymarin on the plasma membranes permeability is linked to the modifications of phospholipids and cholesterol (membrane lipids) which interprets that silymarin may implicate with the secretion of lipoproteins and hepatic enzymes in the blood^54^. Additionally, silibinin can reduce the glycerol integration into lipids of the hepatocytes^55^. While the elevation of the TP levels was attuned by inducing the gene expression of the protein during the silymarin administration^56^. Mereish et al*.* showed that silymarin had an antagonistic effect against the production of the total lipids and triglycerides in the liver of rats injected with CCl_4_^55,57,58^.

Moreover, Treat-CCl_4_ and Prot-CCl_4_ groups showed significant decreases in the relative gene expressions of NF-кB, TGF-β, TNF-α, IL-6, and COX-2 in the hepatic tissues compared to the intoxicated group (Fig. 4). Silymarin hinders the translocation of NF-кB by preventing its activation, thereby switching off the expression and productions of TGF-β, TNF-α, interferon-gamma, IL-2, IL-4, and COX-2 genes. The reduction in the TNF-α gene expression ceases the IL-6 transcription, as shown in Fig. 4. The drop in NF-κB, TGF-β, TNF-α, COX-2, and IL-6, repairs the defects which happened to the liver due to the administration of CCl_4_.

In the current experiment, we found that CCl_4_ decreased serum irisin levels, and this might have contributed to the increased level of inflammation and OS^59,60^. Our study showed that there are a negative correlation between irisin and MDA, NF-κB, and TNF-α in CCl_4_-intoxicated group. This is evidenced by exogenous irisin treatment decreased inflammatory intermediates including IL-6 and TNF-α that are controlled by NF-κB, indicating that there is a negative correlation between irisin level and the level of this cytokines^14,61^. Meanwhile, the level of irisin was improved in the treated and protected groups, which may be due to the antioxidant powers of silymarin^62^. The positive effect of silymarin in achieving normal hepatic function is through irisin production since irisin is intricate with lipid metabolism, and the inflammatory reaction encouraged through AMPK-NF-κB pathway^63–65^. In addition, it was found that the extracts of Silymarin have positive effects on hepatic PGC-1α mRNA expression of diabetic rats, PGC-1α is a main promoter of mitochondrial biogenesis, including ROSdetoxification and irisin production^61,62^. Li et al*.*^66^ suggested that low circulating irisin levels lead to non-alcoholic fatty liver and that Nicotinamide adenine dinucleotide + boosting therapy improves non-alcoholic fatty liver disease via stimulating a novel Fndc5/irisin. Furthermore, a significant reduction in levels of FNDC5/irisin has been indicated in both liver tissue and serum of diabetic rats^67^. In addition, since irisin is expressed and secreted by many tissues, the elevated serum irisin level in this research may be due to the positive impacts of silymarin on the hepatic cells.

## Conclusion

The present experiment indicates the laboratory results of the protective role of silymarin against OS/NSS and liver inflammation induced through the OS induced by xenobiotics. Irisin could be a mediator by which silymarin could induce its hepatoprotective effects by suppressing OS and inflammatory factors. Silymarin pharmaceutical products are struggling seriously to challenge the synthetic drug’s expensive costs, risk factors, and ineffective. Further studies may be performed to test the efficacy of exogenous irisin treatment in hepatic damage induced by xenobiotics and to illustrate the involved mechanisms.

## Data Availability

The datasets used and/or analyzed during the current study available from the corresponding author on reasonable request.
